# Mind the “Happiness” Gap: The Relationship Between Cohabitation, Marriage, and Subjective Well-being in the United Kingdom, Australia, Germany, and Norway

**DOI:** 10.1007/s13524-019-00792-4

**Published:** 2019-07-09

**Authors:** Brienna Perelli-Harris, Stefanie Hoherz, Trude Lappegård, Ann Evans

**Affiliations:** 1 Department of Social Statistics and Demography and Centre for Population Change, School of Social, Economic, and Political Science, University of Southampton, Southampton, SO17 1BJ United Kingdom; 2 Centre for Population Change, University of Southampton, Southampton, United Kingdom; 3 Department of Sociology and Human Geography, University of Oslo, Oslo, Norway; 4 School of Demography, Research School of Social Sciences, Australian National University, Canberra, Australia

**Keywords:** Marriage, Cohabitation, Subjective well-being, Cross-national comparison, Life satisfaction

## Abstract

Many studies have found that married people have higher subjective well-being than those who are not married. Yet the increase in cohabitation raises questions as to whether only marriage has beneficial effects. In this study, we examine differences in subjective well-being between cohabiting and married men and women in midlife, comparing the United Kingdom, Australia, Germany, and Norway. We apply propensity score–weighted regression analyses to examine selection processes into marriage and differential treatment bias. We find no differences between cohabitation and marriage for men in the United Kingdom and Norway, and women in Germany. However, we do find significant differences for men in Australia and women in Norway. The differences disappear after we control for selection in Australia, but they unexpectedly persist for Norwegian women, disappearing only when we account for relationship satisfaction. For German men and British and Australian women, those with a lower propensity to marry would benefit from marriage. Controls eliminate differences for German men, although not for U.K. women, but relationship satisfaction reduces differences. Overall, our study indicates that especially after selection and relationship satisfaction are taken into account, differences between marriage and cohabitation disappear in all countries. Marriage does not lead to higher subjective well-being; instead, cohabitation is a symptom of economic and emotional strain.

**Electronic supplementary material:**

The online version of this article (10.1007/s13524-019-00792-4) contains supplementary material, which is available to authorized users.

## Introduction

A large number of studies have focused on subjective well-being^1^ (see Diener 2000; Helliwell 2003; Kahneman et al. 1999), and many have found that married people have higher subjective well-being (Mikucka 2016; Stutzer and Frey 2006; Waite and Gallagher 2000). Yet the increase in cohabitation—not just as a prelude to marriage but also as an alternative partnership type and an accepted setting for parenthood (Perelli-Harris et al. 2012)—raises questions as to whether only marriage has beneficial effects. Given that cohabitation shares many of the same characteristics as marriage—for example, intimacy, emotional and social support, and joint residence—cohabitors may have similar well-being to those who are married (Soons et al. 2009; Zimmerman and Easterlin 2006).

One of the key issues when analyzing the relationship between partnership status and subjective well-being (SWB) is selection. Cross-sectional studies have often recognized the inability to disentangle selection and causality (e.g., Lee and Ono 2012; Soons and Kalmijn 2009). Longitudinal studies tend to use fixed-effects (FE) models, which focus on within-individual transitions in partnership status, a process usually occurring in younger adulthood and covering a relatively short period of the life course (Kalmijn 2017; Musick and Bumpass 2012; Soons et al. 2009). These studies did not examine the long-term effects of partnership in midlife, after the majority of individuals have made decisions about marriage and childbearing. Midlife^2^—after women’s prime reproductive period, and when being married may influence one’s identity and well-being—is an understudied part of the life course (Lachman 2015). At this point in life, the initial boost in happiness may have declined (Soons et al. 2009; Zimmermann and Easterlin 2006), and raising children may confound SWB (Balbo and Arpino 2016; Margolis and Myrskylä 2015).

FE models also do not directly consider individuals who do not experience a change in partnership status: that is, those who cohabit and never marry. Thus, we cannot tell whether certain groups—for example, those living in a coresidential partnership who have a low propensity to marry—would benefit if they married rather than cohabited; or alternatively, whether the benefits to marriage may be more pronounced for those who have a higher propensity to marry. Given the interest in marriage promotion policies, targeting low-income individuals in countries such as the United Kingdom, it is important to examine whether those unlikely to marry would be happier if they did marry. To address these selection processes, we use propensity score–weighted regression analysis to pinpoint how early life and/or current conditions influence conditions in midlife. This method allows us to address baseline bias and differential treatment bias (Morgan and Winship 2014), which occur when the link between marriage and well-being varies across subgroups.

Cross-sectional research has found a “happiness gap” between cohabitation and marriage in most countries, but the size of the gap appears to vary and may be linked to the acceptance and prevalence of cohabitation in a society (Soons and Kalmijn 2009) or gender context and religious norms (Lee and Ono 2012). However, it is unclear whether the long-term effects of selection in different countries operate similarly, especially because union duration, childbearing experience, and meanings of cohabitation differ across countries (Hiekel et al. 2014; Perelli-Harris et al. 2014). In addition, the heterogeneity of treatment effects may vary, indicating that marriage has different benefits for different groups, depending on the country. Here we compare the association between partnership type and SWB in the United Kingdom, Australia, Germany, and Norway, which have experienced substantial increases in cohabitation over the past few decades but have different family policies (Perelli-Harris and Sánchez Gassen 2012) and cultural orientations toward marriage (Perelli-Harris et al. 2014). Each of these contexts leads us to predict a certain relationship between partnership status and SWB.

This study addresses a number of gaps in the literature. First, we provide new insights into selection due to childhood background and current characteristics for the association between union type and SWB. Second, we examine whether marriage may be especially advantageous for partnered individuals who have a lower or higher propensity to marry. Third, we analyze the extent to which the relationship between partnership type and SWB varies by country and gender. More broadly, analyzing how cohabitation differs from marriage for individuals’ well-being will contribute to our understanding of the meaning and consequences of cohabitation as well as the extent to which these meanings differ across contexts.

## Theoretical Background

A large body of research has investigated the beneficial aspects of marriage for well-being (for reviews, see Nelson-Coffey 2018; Waite and Gallagher 2000). These studies posited that married partners benefit from sexual and emotional intimacy, companionship, and daily interaction (Kamp Dush and Amato 2005; Umberson et al. 2010). Spouses help each other cope with stress by providing social and emotional support. Recognition from a spouse may provide symbolic meaning in life (Umberson et al. 2010). Additionally, sharing a household can lead to economies of scale, and married spouses could profit from a larger friendship and kin network (Ross and Mirowsky 2013; Umberson and Montez 2010). All these mechanisms could enhance SWB.

Nonetheless, given dramatic social change over the past decades, the benefits to marriage may be declining (Liu and Umberson 2008). A recent study comparing 87 countries found that the life satisfaction advantage of married men compared with unmarried men has waned over the last three decades, suggesting that marriage has become less advantageous (Mikucka 2016). This decline may be partially due to the increase in cohabitation, especially in high-income countries. Cohabitation may be taking on much of the form and function of marriage (Cherlin 2004), especially as cohabiting unions become longer and involve children. Similar to married couples, cohabiting couples share a household and may benefit from similar intimacy, support, care, and family networks. Normative expectations to marry have become weaker, and the tolerance for nonmarital arrangements has increased in most countries (Treas et al. 2014).

A large body of research, however, has found that cohabitors often differ from married couples. Across countries, cohabitors have lower second birth rates (Perelli-Harris 2014), are less likely to pool incomes (Gray and Evans 2008; Lyngstad et al. 2010), have lower relationship quality (Wiik et al. 2012), and are more likely to dissolve their relationships (Galezewska et al. 2017), even if they have children (Musick and Michelmore 2018). Qualitative research from Europe and Australia has suggested that many still think of cohabitation as a less-committed type of union than marriage and instead oriented toward freedom and independence (Perelli-Harris et al. 2014). Marriage may thus still be desired by most people but more as a cultural ideal or status symbol.

Recently, most scholars have used FE models to examine whether cohabitation is similar to marriage in increasing SWB. This approach allows the testing of set-point theory, which posits that individuals have a baseline level of happiness that cannot be permanently modified by life events, such as union formation. This theory has been tested in a range of settings, and the findings support a positive effect of marriage and cohabitation on SWB (Musick and Bumpass 2012; Soons et al. 2009; Zimmermann and Easterlin 2006), with cohabitation having a weaker effect (Kalmijin 2017). Some studies, however, have questioned set-point theory and found that different model specifications can result in long-term improvements for marriage (Anusic et al. 2014). Overall, however, most studies indicated that, on average, marriage provides a boost to well-being, with cohabitation providing a weaker boost, and individuals return to original happiness levels in the long term.

### Selection Processes

Selection processes—also referred to as *baseline bias* (Morgan and Winship 2014)—select people into marriage and may be responsible for higher SWB in midlife. These processes can begin early in childhood and continue into adulthood (Elo 2009; Kuh et al. 2004; Umberson et al. 2010). For example, parents’ education and socioeconomic status (SES) are strongly associated with adult life satisfaction (Frijters et al. 2014; Layard et al. 2014), but can also influence decisions about cohabitation and marriage, especially around the time of a first birth (Koops et al. 2017; Wiik 2009). Parental divorce in childhood may have long-term effects on future SWB, both emotionally and financially (Amato 2010), and lead the children of divorced parents to choose cohabitation over marriage for their first relationship (Perelli-Harris et al. 2017). Thus, any positive association between marriage and SWB may not be due to the benefits of marriage but instead childhood conditions and experiences, which may both select people into marriage and lead to higher SWB.

Prior research has consistently found that current conditions, such as income and education, are also important for understanding SWB (e.g., Diener et al. 2009; Layard 2011). Unemployment has a persistent negative effect on SWB, especially for men (Clark et al. 2008). Household income and partner’s education and employment status may be particularly important for women if they are not working. In addition, people with higher education and better economic prospects are more likely to marry (Kalmijn 2013; Sweeney 2002) and stay married (Matysiak et al. 2014). Another key indicator of SWB in adulthood is poor health (Binder and Coad 2013), which can lead to poor relationship quality and potentially influence marriage decisions. Unlike selection in childhood, selection in adulthood could be confounded with partnership formation and happiness; for example, a woman could drop out of the labor market after marriage, or unhappy people could be more likely to lose their job.

### Differential Treatment Bias

Selection processes result in individuals having a different propensity to cohabit or marry, which can produce *differential treatment bias* (Morgan and Winship 2014). The effect of the treatment, in this case marriage, can vary based on selection characteristics. As we discuss earlier, most prior research has shown that marriage is selective of those who grew up in stable married-parent families with higher SES, and cohabitation is selective of those raised by single mothers or low-income parents (Wiik 2009). Thus, the benefits of marriage over cohabitation may be differentially distributed according to these background characteristics (Su et al. 2015). The differential treatment effect may result in marriage affecting SWB through different potential mechanisms.

#### High Propensity to Marry, Positive SWB

Individuals with a high propensity to marry may be more likely to have higher SWB if they marry, because they may be more affected by social norms and expectations to marry, especially because marriage can be considered an expression of status (Cherlin 2010). They may benefit more from the legal security and access to courts that official marriage provides when property or resources are combined, especially for couples who have children. Thus, marriage may protect their standard of living and provide a sense of security in case of divorce (Perelli-Harris et al. 2014).

#### High Propensity to Marry, Negative/Neutral SWB

Those with a higher propensity to marry may not benefit more from marriage than cohabitation, because they have the emotional, social, and financial resources to provide a positive outlook regardless of marital status.

#### Low Propensity Positive SWB

People with a lower propensity to marry may benefit more from marriage, because it is recognized not only legally but also by family, friends, and the community, who may then provide greater social support (Marcussen 2005; Umberson and Montez 2010). Rather than symbolizing social status, marriage may signify having achieved stability and protection. Married people may also have greater trust in the long-term prospects of their relationship given that marriage is usually intended for life.

#### Low Propensity to Marry, Neutral/Negative SWB

Marriage may not be advantageous to those with a low propensity to marry, if individuals have no desire to marry their partners. Those from a disadvantaged background are more likely to have partners less suitable for marriage—for example, because they are unemployed or have fewer resources and greater debt. They may be happier cohabiting because of the high perceived risk of divorce and its associated costs.

## Gender Differences

Although some prior studies have found few gender differences in the effects of marriage on psychological well-being (e.g., Williams 2003), men and women may receive different benefits from being in marriage compared with cohabitation (Liu and Umberson 2008), and these benefits may differ by the propensity to marry. Previous studies have argued that marriage provides men with greater social recognition and support, thereby positively influencing their well-being (Ross et al. 1990). Men may benefit from the status of marriage more than women, particularly in the workplace (Killewald and Gough 2013). On the other hand, if men benefit more from emotional support and regular sexual intimacy, cohabitation may provide advantages similar to those of marriage. Women could benefit more from marriage because of the higher economic resources and legal protection that provides them with a sense of safety (Waite 1995), which are especially important when bearing and raising children. Women may also feel a sense of satisfaction from the wedding and achieving normative social aspirations (Berrington et al. 2015). Nonetheless, women may prefer cohabitation if they oppose the traditional, patriarchal constraints of marriage. Disadvantaged women may not want to marry unsuitable partners who cannot achieve a certain economic bar (Edin and Reed 2005; Lichter et al. 2006; Smock et al. 2005). In such cases, marriage for women may be more detrimental to SWB than cohabitation.

### Cross-National Differences

Welfare state context, family policies, and cultural attitudes shape partnership formation and may influence the association between partnership formation and SWB. Selection processes may also differ across countries, which may in turn produce heterogeneous treatment effects, with some groups benefiting more from marriage than others. Table 1 outlines how welfare state, legal system, social norms, and strength of selection would predict differences in SWB by partnership on average and by those with a high or low propensity to marry in each country.

**Table 1:** Brief description of welfare state, legal status of cohabitation, social norms, and social selection in each country (see the text for references): Summary of average and differential expectations for men and women

	Welfare State	Legal Status of Cohabitation	Social Norms	Social Selection Into Midlife Cohabitation	Average Expectations for Men	Differential Expectations for Men ^a^	Average Expectations for Women	Differential Expectations for Women ^a^
United Kingdom	Liberal welfare state	Inferior/ignored in most policy areas	Cohabitation is accepted, but marriage is often preferred.	Strong based on disadvantage	Significant difference between cohabitation and marriage, but eliminated after selection taken into account	HP, neutral SWBLP, positive SWB	Significant difference between cohabitation and marriage, but eliminated after selection taken into account	HP, neutral SWB LP, positive SWB
Australia	Liberal welfare state, with higher benefits	Equivalent to marriage (after 0.5 year or with children)	Cohabitation is accepted, but marriage is often preferred.	Strong based on disadvantage	Significant difference between cohabitation and marriage, but eliminated after selection taken into account	HP, neutral SWBLP, positive SWB	Significant difference between cohabitation and marriage, but eliminated after selection taken into account	HP, neutral SWB LP, neutral SWB
Germany	Conservative welfare state	Inferior/tax advantages to marriage, especially if one partner earns more	Marriage is usually ideal in West; cohabitation is much more accepted in East.	Weak based on disadvantage	Significant difference between cohabitation and marriage	HP, neutral SWBLP, positive SWB	Significant difference between cohabitation and marriage	HP, neutral SWB LP, positive SWB
Norway	Social-democratic welfare regime	Mostly equivalent to marriage (after two years or with children)	Cohabitation and marriage are equal, but marriage is sometimes preferred.	Weak based on disadvantage	No difference between cohabitation and marriage	HP, neutral SWBLP, neutral SWB	No difference between cohabitation and marriage	HP, neutral SWB LP, positive SWB

^a^HP = high propensity to marry. LP = low propensity to marry.

The United Kingdom has been classified as a liberal welfare state (Esping-Andersen 1990) with a minimal safety net and a prioritization of means-tested benefits. In England, the Conservative party promoted marriage as an institution, and the current UK government legislated tax breaks for married couples in 2013 (BBC 2015). Lawyers have described the legal situation for cohabitors as chaotic, with cohabiting couples receiving little access to family courts upon separation and receiving no inheritance rights upon death of a partner (Barlow 2014). Cohabitation is strongly selective of disadvantage (Perelli-Harris et al. 2010), and the means-tested welfare system may provide further disincentives to marry. Thus, the lack of rights for cohabitors and the social norms favoring marriage should on average make marriage more advantageous than cohabitation. However, differences between cohabitation and marriage should disappear after selection is taken into account. Nonetheless, both men and women in partnered unions with a low propensity to marry should have higher SWB if married (low propensity, positive SWB), because marriage would provide them with a greater sense of security and stability, especially important when the welfare state does not provide sufficient support.

Although Australia is often classified as a liberal welfare state, it has higher levels of benefits (Arts and Gelissen 2002), which may result in women being less dependent on marriage. The state also allows cohabiting couples the same access to family courts as married couples upon union dissolution and similar rights to inheritance (Evans 2015). Nonetheless, highly educated individuals are more likely to be married than lower-educated individuals (Evans 2015; Heard 2011). Thus, we expect stark differences in SWB between cohabitation and marriage on average but also that these differences will be eliminated after taking selection into account. Given the strong state support for cohabiting couples, women with a low propensity to marry would not be better off if married (low propensity, neutral/negative), but men with a low propensity to marry would still benefit from the perceived social status and stability achieved through marriage (low propensity, positive SWB).

Germany has a traditional welfare state with a generally conservative view on families (Esping-Andersen 1990). Marriage was enshrined in the Constitution, and the German state directly promotes marriage with tax incentives, especially relevant when couples have children, and the mother drops out of the labor force (Perelli-Harris and Sánchez Gassen 2012). Most laws related to health insurance, inheritance, and property regulation favor married couples. Given the normative and legal privileging of marriage, we expect on average that SWB will be higher among those who are married. On the other hand, tax benefits to marriage apply only to couples in which the man earns substantially more than the wife; dual-earner couples would be just as well off if they cohabit. Thus, we expect that marriage will provide fewer benefits for both men and women with a high propensity to marry (high propensity, neutral/negative SWB) because they would be happy regardless of marriage. Men and women with a lower propensity to marry, however, would benefit more from marriage, because it would provide them with a greater sense of security and stability (low propensity, positive SWB).

Norway’s social-democratic welfare state, with its emphasis on gender equality and individual autonomy (Esping-Andersen 1990), may have facilitated the increase in cohabitation (Lappegård and Noack 2015). Norwegian policies tend to support dual-earner families by providing parental leave and public childcare; but the tax and transfer system, which focuses on individuals, may lower the incentives for couples to marry (Perelli-Harris and Sánchez Gassen 2012). The legal system has also harmonized many rights and responsibilities between cohabiting and married people, especially for those with children and in long-term unions; however, inheritance rights are still reserved for married spouses. Overall, the general tolerance of cohabitation in Norway (Treas et al. 2014) and high levels of nonmarital childbearing suggest that on average, cohabiting and married individuals should have similar levels of SWB, regardless of their likelihood to marry. On the other hand, prior research has indicated that low-educated women are more likely to give birth within cohabitation than highly educated women (Perelli-Harris et al. 2010), indicating that cohabitation is selective of disadvantage. Thus, we expect that women with a low propensity to marry would benefit from marriage because of the increased social support and family networks that marriage would provide to disadvantaged women (low propensity, positive SWB).

## Data, Methods, and Measures

### Data

We employ four harmonized data sets, which are the best suited in each country for studying the effects of partnership status in midlife. For the United Kingdom, we use the UK Household Longitudinal Study (UKHLS), a nationally representative, household-based longitudinal survey (University of Essex 2018). The survey started in 2009 with approximately 51,000 individuals and is conducted annually. Our sample comes from the fourth wave conducted in 2012/2013, with a total of 47,157 individuals surveyed. Our sample aged 38–50 includes 8,941 individuals who answered the SWB and partnership questions.

The Australian data come from the Household, Income and Labour Dynamics in Australia (HILDA), a nationally representative, household-based longitudinal survey. The survey started in 2001 and annually interviews all adults over age 15 (13,969 individuals) in the selected households. The sample expanded with a general top-up at Wave 11 in 2012; in 2013 (our analysis year), 13,536 individuals were interviewed. After excluding those not aged 38–50 and who did not answer the SWB and partnership questions, our sample comprises 3,787 individuals.

For Germany, we use the Socio-Economic Panel (SOEP), a representative longitudinal study of households, with all household members interviewed annually (from age 15). SOEP began in 1984 with 12,290 individuals. Apart from the inclusion of participants from the former East-German state after German reunification, this study had several refreshment samples over its 30-year duration in order to assure national representation. Our sample comes from the 2013 wave, which surveyed 24,113 individuals, 8,830 of whom were aged 38–50.

We also use the Norwegian Generations and Gender Survey (GGS) because no equivalent panel surveys study partnerships in midlife in Norway, and cohabitation among couples without children has been only recently recorded in the registers. The GGS is a nationally representative cross-sectional survey of respondents aged 18–79 and has information that can approximate our longitudinal design. It includes 15,114 individuals interviewed by telephone and a self-administered questionnaire (SAQ) in 2007, combined with data from administrative records. After excluding those who did not answer the SWB or partnership questions, we include 2,785 individuals aged 38–50 in our sample.

### Methods

**Fig. 1:**
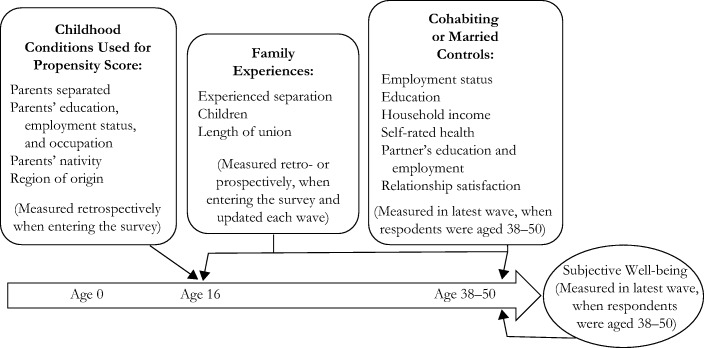
Analytic approach

In this study, we use propensity score–weighted regression analysis to address both types of selection: baseline bias and differential treatment bias (e.g., Su et al. 2015). This approach has the advantage of addressing selection while incorporating contemporaneous factors that may influence SWB. Figure 1 presents our analytical approach, depicting the variables from childhood (0–16) through to midlife (38–50). Childhood conditions are used in the propensity score and included as controls in the weighted regression models. Prior family experiences, captured for the age range 16–50, and current factors measured at the time of the interview are included as controls in the weighted regression models.

In the first step of the analysis, we estimate propensity scores using logistic regression for the probability of selection into the treatment group—marriage—based on a set of observed characteristics (Rosenbaum and Rubin 1983). See Table A1 in the online appendix for the logistic regression results for each country. In the second step, the propensity scores are used as weights in regression analyses (Morgan and Todd 2008). Through weighting, the treatment and control group become comparable. For example, cohabiting individuals who are similar to married individuals with respect to background characteristics are up-weighted, and cohabitors with features dissimilar to those of the married are down-weighted. We obtain three weights that function as conditional predicted probabilities of being married at midlife. The first weight is the average treatment effect (ATE), which estimates the effect of marriage on SWB for the entire sample, controlling for selection and other characteristics (Eq. (1a)). The second weight is the average treatment effect on the treated (ATT), which estimates the effect of marriage for those with a high propensity to marry (Eq. (1b)). The third weight, the average treatment effect of the control (ATC), shows the effect that marriage would have on SWB for those with a low propensity to marry relative to cohabiting (Eq. (1c)).

1a $$ {\displaystyle \begin{array}{l}\mathrm{For}\kern0.5em {d}_i=1:{w}_{i, ATE}=\frac{1}{{\hat{p}}_i}\\ {}\mathrm{For}\kern0.5em {d}_i=0:{w}_{i, ATE}=\frac{1}{1-{\hat{p}}_i}\end{array}} $$

1b $$ {\displaystyle \begin{array}{l}\mathrm{For}\kern0.5em {d}_i=1:{w}_{i, ATT}=1\\ {}\mathrm{For}\kern0.5em {d}_i=0:{w}_{i, ATT}=\frac{{\hat{p}}_i}{1-{\hat{p}}_i}\end{array}} $$

1c $$ {\displaystyle \begin{array}{l}\mathrm{For}\kern0.5em {d}_i=1:{w}_{i, ATC}=\frac{1-{\hat{p}}_i}{{\hat{p}}_i}\\ {}\mathrm{For}\kern0.5em {d}_i=0:{w}_{i, ATC}=1.\end{array}} $$

Weighting seeks to make the treatment and control groups comparable, or balanced, in terms of certain characteristics. We test how well the data are balanced by estimating the average standardized mean difference between treatment and control groups for all categorical covariates, and the standardized difference in standard deviations for continuous covariates (see Morgan and Todd 2008). Table A2 in the online appendix shows that the weights successfully balance the data, as shown in the improved average standardized mean and standard deviation. Estimating the same regression models (available on request) for sample members of the treatment group who have a counterpart in the control group—*cases of common support* (Morgan and Todd 2008)—supports our initial interpretation. We also use a combination of propensity score weighting with covariate adjustment, which can correct a small imbalance when the propensity score weighting may not have sufficiently balanced the sample (Morgan and Todd 2008). Although propensity score analyses are effective at removing bias caused by observed variables, omitted variables cannot be considered in this adjustment.

In a third step, we estimate ordinary least squares (OLS) regressions using the three propensity weights for men and women in each country (Eq. (2)). Although the estimation of the propensity scores requires complex modeling, the final analysis is generally straightforward. *Y*is level of SWB; *D* is the treatment variable (being married); δ̂ is the estimated effect of *D* on *Y*, adjusted for **X**; and **X** is a vector of observed variables that are thought to determine *D* and *Y*. We first estimate the average effect of being married on SWB with regressions that applied the ATE weights. We next assess causal effect heterogeneity by comparing the results from ATT- and ATC-weighted regressions. In other words, we compare the effect of marriage for those with a high propensity to marry (ATT) with the effect of marriage on those with a low propensity to marry (ATC).

2 $$ Y=\upalpha \hat{\mkern6mu} +\updelta \hat{\mkern6mu} \mathrm{D}+\mathbf{X}\upbeta \hat{\mkern6mu} +\upvarepsilon . $$

### Measures

We harmonize the variables in each survey, although region remains context-specific. Missing observations for independent variables are imputed using the mi impute command in Stata 13.0.

#### Dependent Variable

In all countries, SWB is measured with a single item in the latest wave analyzed.^3^ The responses are recorded on an 11-point scale ranging from 0 = completely dissatisfied to 10 = completely satisfied with life, except in the United Kingdom, where the scale ranges from 1 = completely dissatisfied to 7 = completely satisfied. The single-item life satisfaction measure is widely used in demographic research (e.g., Balbo and Arpino 2016) and has similar psychometric characteristics to multiple-item scales (Cheung and Lucas 2014).

#### Age

All models include a yearly control for age, given that the age range is 38–50. Other specifications of the age range do not alter the results.

#### Childhood Characteristics

We distinguish three types of childhood background characteristics important for union formation and well-being: (1) region, and parent’s nativity; (2) family structure in childhood; and (3) parental SES. We choose the most relevant measure of region for each country.

Large regional differences exist across the United Kingdom, and we control for four major regions: (1) Scotland, Ireland, and North England; (2) Midlands and Wales; (3) South West England; and (4) South East England. In Australia, we include a control only for differences between rural and urban areas: prior studies have indicated these are the most salient for understanding geographic disparities (Monnat and Beeler Pickett 2011). In Germany, the East-West divide is particularly important for family formation. In eastern Germany, nearly two-thirds of children were born in cohabitation; in western Germany, only one-third were born in cohabitation (Perelli-Harris et al. 2012). Because of these regional distinctions and migration from the East to the West after reunification, we control for the following four categories: (1) West Germany; (2) East Germany stayed; (3) East moved to West Germany; and (4) born outside Germany. In Norway, we control for four regions: (1) Oslo area; (2) East; (3) South and West; and (4) Mid and North.

In all countries, we include indicators for whether the respondent and the respondent’s parents were born in the country. Family structure in childhood includes a dummy variable for whether the respondent lived with both parents at age 15 (Norway) or up to age 16 (United Kingdom, Australia, and Germany). SES of parents includes mother’s and father’s education (low, medium, and high), whether mother worked, and father’s occupation (low, medium, high, and not employed) when respondent was aged 14 or 15.

#### Partnership and Childbearing Experiences

As we discuss earlier, cohabiting partnerships could be very similar to marital partnerships, especially with regard to union duration and childbearing behavior. These experiences could also be endogenous to choice of partnership type. Longer union duration often signals a deeper investment in the relationship (Lyngstad et al. 2010), which could reduce differences between cohabitation and marriage. Current union duration is included as a linear variable.^4^ Parenthood also directly affects SWB, but results from previous studies are mixed, indicating that children can have a positive or negative impact (Aassve et al. 2015; Balbo and Arpino 2016; Myrskalä and Margolis 2014; Pollmann-Schult 2014). Partnership and childbearing histories were collected in different ways in each survey. In the UKHLS, HILDA, and GSOEP, they were collected retrospectively in the first wave of the survey and updated during each additional panel. In the Norwegian GGS, the histories were collected retrospectively at the main wave of the survey (2007). Here, childbearing history is represented with three categories: (1) respondent has no child; (2) respondent has at least one child, but not with current partner; and (3) respondent has at least one child with current partner.^5^ Other specifications of childbearing history, including total number of children, do not change the main effects of partnership status on SWB.

The experience of union dissolution is another important control because it can have long-term effects on health and mental well-being, even if individuals repartner (Amato 2010; Hughes and Waite 2009). Separation can also influence decisions about repartnering: people who have separated are more likely to choose cohabitation for subsequent partnerships (Galęzewska et al. 2017). A dummy variable indicates whether the respondent ever experienced separation and/or divorce. We also test models restricted to repartnered individuals with children, and the results do not change substantially.

Finally, relationship satisfaction is another key factor that may be very important for mediating the effect of marriage on well-being. Any association between marriage and SWB may not be due to marriage itself but instead may be due to married people, on average, having higher relationship satisfaction, which is highly correlated with SWB. Thus, some individuals in cohabiting relationships may have similar levels of well-being to those in marital relationships if their relationship provides them with similar levels of satisfaction. On the other hand, relationship satisfaction is most likely endogenous to both marriage and SWB. People who are more satisfied with their relationship are more likely to have higher levels of SWB (Kamp Dush and Amato 2005), and happier couples are more likely to marry (Gustavson et al. 2016). In addition, cohabitors are usually less satisfied with their relationships than married individuals (Wiik et al. 2012). We include relationship satisfaction in our models to control for the potential similarities between cohabitation and marriage, but relationship satisfaction could also be interpreted as a mediator between marriage and SWB, and it may be a proxy for the decision to marry. Relationship satisfaction is measured with a scale from 0 (very unhappy with relationship) to 10 (very happy with relationship) for Australia and Norway. For the United Kingdom, the scale ranges from 0 to 7, and we rescale it to be similar to the other countries. For Germany, because relationship satisfaction was not asked, we control for satisfaction with family life, measured on a scale of 0–10.

#### Current Situation

We control for contemporaneous factors measured at the time of the most recent survey, which could be considered endogenous but have been found to be very important for SWB (Kamp Dush and Amato 2005). We include self-rated health measured on a five-level scale (from 1 = poor to 5 = excellent). The socioeconomic background of the person is represented by education (low, medium, or high), employment status (employed, out of the labor force, or unemployed), and household income (quintiles). Partner’s education is also measured in three categories (low, medium, or high); however, partner’s employment status is a dummy variable (employed or out of the labor force).

## Results

### Descriptive Statistics

Table 2 presents (1) the percentage of individuals living with and without a partner for the entire sample and (2) the percentage married and cohabiting among those who are partnered. It also shows mean SWB (with confidence intervals) by partnership type. Immediately, we see large significant differences in SWB between the partnered and unpartnered in all countries. Differences between cohabitation and marriage, however, are significant only in the United Kingdom and Australia. Table 3 presents the key independent variables that may explain differences between the two relationship types. Because Table 3 indicates gender difference in levels of SWB, we perform separate analyses by gender.

**Table 2:** Percentage and number of those partnered or unpartnered, and married or cohabiting, mean subjective well-being, and 95 % confidence intervals (CI), men and women aged 38–50

	United Kingdom		Australia		Germany		Norway	
	% (*n*)	Mean (95 % CI)	% (*n*)	Mean (95 % CI)	% (*n*)	Mean (95 % CI)	% (*n*)	Mean (95 % CI)
Partnered	67	7.44	69	7.80	80	7.45	74	8.35
	(6,006)	(7.39,7.49)	(2,629)	(7.75,7.85)	(7,085)	(7.41,7.49)	(2,051)	(8.30,8.42)
Unpartnered	33	6.55	31	7.23	20	6.49	26	7.84
	(2,935)	(6.45,6.65)	(1,158)	(7.13,7.33)	(1,745)	(6.40,6.58 )	(734)	(7.70,7.97)
Total *N* of Sample	8,941	7.21	3,787	7.64	8,830	7.21	2,785	8.22
		(7.16,7.26)		(7.59,7.69)		(7.17,7.24)		(8.14,8.30)
Married	83	7.48	87	7.84	88	7.46	84	8.43
	(4,988)	(7.42,7.53)	(2,288)	(7.78,7.89)	(6,269)	(7.42,7.50)	(1,727)	(8.37,8.49)
Cohabiting	17	7.25	13	7.48	12	7.35	16	8.24
	(1,018)	(7.12,7.37)	(341)	(7.32,7.64)	(816)	(7.24,7.46)	(324)	(8.09,8.39)
Total *N* of Partnered	6,006	7.43	2,629	7.80	7,085	7.44	2,051	8.40
		(7.39,7.49)		(7.45,7.85)		(7.40,7.47)		(8.33,8.47)

*Source:* Own calculations using UKHLS (United Kingdom), HILDA (Australia), SOEP (Germany), and GGS (Norway).

**Table 3:** Descriptive statistics for cohabiting (COH) and married (MAR) men and women in midlife

	United Kingdom				Australia				Germany				Norway			
	Men		Women		Men		Women		Men		Women		Men		Women	
	COH	MAR	COH	MAR	COH	MAR	COH	MAR	COH	MAR	COH	MAR	COH	MAR	COH	MAR
Subjective Well-being	7.3	7.4	7.2	7.5	7.4	7.8	7.6	7.9	7.1	7.5	7.5	7.4	8.2	8.3	8.1	8.5
Mean/SD	2.1	2.2	2.4	2.3	1.4	1.3	1.7	1.3	1.5	1.5	1.5	1.5	1.2	1.3	1.7	1.3
Family Behavior																
Union duration in years	11.2	16.5	11.8	18.5	9.8	14.9	12.6	16.7	8.5	21.2	8.7	21.8	11.7	17.6	13.9	20.2
Mean/SD	7.7	6.7	8.2	6.9	7.4	7.2	8.2	7.6	6.2	11.5	5.7	10.1	6.5	6.3	6.7	6.3
Ever separated (%)																
No previous cohabiting union	36	75	29	76	44	88	47	86	67	62	78	62	53	78	53	77
Separated/divorced	64	25	71	24	56	12	53	14	33	38	22	38	47	22	47	23
Children with partner (%)																
No children	50	32	46	38	37	7	32	5	44	9	30	7	23	6	18	4
Child with previous partner	15	5	18	4	21	4	21	6	29	20	43	20	24	11	18	11
Child with current partner	36	63	36	58	42	89	47	89	27	71	27	73	53	84	64	85
Relationship satisfaction^a^	6.6	7.0	6.5	6.8	7.5	8.1	7.5	7.9	7.9	8.3	7.8	8.1	8.5	8.7	8.2	8.7
Mean/SD	2.1	2.1	2.2	2.2	2.2	1.9	2.3	2.0	1.7	1.5	1.7	1.5	1.5	1.4	2.0	1.4
Childhood Background																
Parental separation^b^																
Yes	29	20	28	20	24	15	23	16	27	13	21	15	11	8	10	7
No	71	80	72	80	76	85	77	84	73	87	79	85	89	92	90	93
Both parents native (%)																
Yes	79	66	77	66	38	57	48	56	79	85	90	90	93	90	94	90
At least one foreign-born	21	34	23	34	62	43	52	44	21	15	10	10	7	10	6	10
Mother’s education (%)																
Low	35	39	43	40	60	53	57	56	20	32	23	30	44	44	48	39
Medium	58	55	51	53	31	33	27	27	71	55	70	58	48	44	44	49
High	7	6	6	7	9	14	18	17	9	13	7	12	8	12	8	12
Father’s education (%)																
Low	35	35	41	37	33	36	38	40	8	16	10	14	33	32	43	28
Medium	55	53	50	52	53	43	44	41	70	65	77	66	56	48	49	48
High	10	12	10	12	14	21	18	19	22	19	13	20	11	20	8	24
Mother’s employment status (%)																
Not employed	32	39	30	32	41	43	42	46	21	28	23	32	33	37	29	31
Employed	68	61	70	68	59	57	58	54	79	72	76	68	67	63	71	69
Father’s employment status (%)																
Not employed	7	6	8	7	8	8	9	9	6	4	8	4	1	3	2	3
Employed	93	94	92	93	92	92	91	91	94	96	92	96	99	97	98	97
Father’s occupation (%)																
Low	59	54	63	57	26	25	28	23	20	28	27	23	74	65	74	63
Medium	11	12	9	10	29	33	36	32	35	39	52	44	23	30	22	31
High	30	34	28	33	45	42	36	45	45	33	21	33	3	6	4	6
Current Situation																
Education (%)																
Low	17	16	20	13	32	21	33	35	5	6	5	8	25	19	29	23
Medium	48	38	40	40	46	42	29	28	67	64	69	63	52	53	36	39
High	35	46	40	47	22	37	38	37	28	30	26	29	23	28	35	38
Household income quintiles (%)																
First	15	9	12	8	18	8	15	8	12	5	8	5	12	8	29	31
Second	21	18	25	15	26	20	23	19	9	11	9	13	14	8	35	30
Third	24	22	21	22	21	21	20	22	20	21	24	18	23	19	20	18
Fourth	20	25	23	27	24	25	27	24	28	38	37	36	29	29	9	13
Fifth	20	26	19	28	11	26	15	27	30	26	22	28	22	36	7	8
Employment status (%)																
Out of labor force	7	4	16	18	12	4	20	21	3	5	4	19	5	5	12	12
Unemployed	8	4	7	3	4	3	2	1	9	4	8	4	1	1	1	1
Employed	85	92	77	79	84	93	78	78	88	91	88	77	94	94	87	87
Self-rated health	3.4	3.5	3.3	3.5	3.4	3.5	3.5	3.5	3.5	3.6	3.4	3.5	3.7	3.7	3.7	3.7
Mean/SD	1.2	1.2	1.2	1.2	1.0	1.0	1.0	0.9	0.9	0.9	0.8	0.9	1.1	1.1	1.2	1.1
Partner’s education (%)																
Low	14	12	19	16	33	33	29	21	8	10	5	6	2	6	13	9
Medium	42	40	47	42	36	29	46	43	72	66	64	64	63	53	65	54
High	44	48	34	42	31	39	25	36	20	24	31	30	35	41	22	37
Partner’s employment status (%)																
Out of labor force	25	23	17	9	25	20	16	7	25	25	13	8	12	12	8	5
Employed	75	77	83	91	75	80	84	93	75	75	87	92	88	88	92	95
Region^c^																
1	33	35	38	34	67	68	61	68	62	58	61	61	15	20	15	20
2	22	19	21	21	33	32	39	32	21	14	25	12	35	27	35	28
3	11	9	11	9	––	––	––	––	3	4	6	3	24	38	27	35
4	34	37	30	36	––	––	––	––	14	24	8	23	26	15	24	17
Total *N*	491	2,253	527	2,735	178	1,084	163	1,204	420	2,921	396	3,348	150	774	174	953
%	18	82	16	84	14	86	12	88	13	86	11	89	16	84	15	85

*Source:* Own calculations with UKHLS, HILDA, SOEP, and GGS; data are weighted.

^a^For Germany, we include satisfaction with family life because relationship satisfaction was not asked.

^b^Parents separated during childhood.

^c^Region in United Kingdom: 1 = Scotland, Ireland, and North England; 2 = Midlands and Wales; 3 = South West England; and 4 = South East England. In Germany: 1 = West Germany, 2 = East Germany stayed, 3 = East moved to West Germany, and 4 = born outside Germany. In Australia: 1 = urban, and 2 = rural. In Norway: 1 = Oslo area, 2 = East, 3 = South and West, and 4 = Mid and North.

### OLS Regressions

Table 4 presents coefficients for marriage relative to cohabitation for OLS regression models of SWB in midlife (full tables available on request). The unweighted column shows the unconditional association between marriage and mean SWB. The ATE column presents the average treatment effect after we apply weights, which can indicate the extent to which selection processes are biasing the results. The next two columns address our research questions about differential treatment bias: ATT refers to the average treatment effect on the treated (those in a partnership with a high propensity to marry), and ATC refers to the average treatment effect on the controls (those in a partnership with a low propensity to marry). The first row includes only controls for partnerships status and age. The second row includes all our control variables.^6^ The third row adds relationship satisfaction (satisfaction with family life in Germany), which is important to examine separately because of endogeneity.

**Table 4:** OLS weighted regression coefficients for the association between marriage and subjective well-being relative to cohabitation at midlife (ages 38–50)

	Men				Women			
	Unweighted	ATE	ATT: High Propensity to Marry	ATC: Low Propensity to Marry	Unweighted	ATE	ATT: High Propensity to Marry	ATC: Low Propensity to Marry
United Kingdom								
(1) Married vs. cohabiting + age	0.086	0.021	0.003	0.101	0.327**	0.289*	0.235	0.283**
	(0.105)	(0.104)	(0.105)	(0.105)	(0.120)	(0.126)	(0.128)	(0.125)
(2) + Childhood characteristics + partnership behavior + person’s and partner’s SES in current year^a^	–0.055 (0.114)	–0.079 (0.118)	–0.093 (0.121)	–0.013 (0.116)	0.225 (0.134)	0.334* (0.150)	0.300 (–0.173)	0.405* (0.145)
(3) + Satisfaction with relationship	–0.136	0.069	–0.182	–0.112	0.132	0.258	0.224	0.279
	(0.112)	(0.114)	(0.117)	(0.113)	(0.129)	(0.147)	(0.149)	(0.140)
Number of observations	3,556				3,262			
Australia								
(1) Married vs. cohabiting + age	0.351**	0.314*	0.310*	0.335**	0.245*	0.181	0.170	0.261*
	(0.109)	(0.131)	(0.134)	(0.120)	(0.116)	(0.127)	(0.127)	(0.132)
(2) + Childhood characteristics + partnership behavior + person’s and partner’s SES in current year^a^	0.211 (0.110)	0.193 (0.126)	0.193 (0.129)	0.199 (0.120)	0.214 (0.116)	0.156 (0.120)	0.147 (0.121)	0.232 (0.123)
(3) + Satisfaction with relationship	0.028	0.018	0.016	0.036	0.056	–0.001	–0.011	0.082
	(0.105)	(0.123)	(0.126)	(0.114)	(0.109)	(0.108)	(0.108)	(0.114)
Number of observations	1,262				1,367			
Germany								
(1) Married vs. cohabiting + age	0.166*	0.127	0.111	0.234**	0.024	-0.035	–0.044	0.037
	(0.085)	(0.095)	(0.100)	(0.078)	(0.090)	(0.101)	(0.103)	(0.096)
(2) + Childhood characteristics + partnership behavior + person’s and partner’s SES in current year^a^	0.134 (0.082)	0.119 (0.099)	0.118 (0.103)	0.130 (0.081)	0.016 (0.089)	–0.032 (0.091)	0.027 (0.091)	–0.094 (0.092)
(3) + Satisfaction with relationship	0.086	0.119	0.124	0.089	0.047	–0.034	–0.029	–0.098
	(0.076)	(0.093)	(0.097)	(0.074)	(0.082)	(0.085)	(0.086)	(0.082)
Number of observations	3,341				3,744			
Norway								
(1) Married vs. cohabiting + age	0.066	0.075	0.073	0.087	0.274*	0.336**	0.341**	0.314*
	(0.114)	(0.103)	(0.104)	(0.105)	(0.110)	(0.119)	(0.121)	(0.125)
(2) + Childhood characteristics + partnership behavior + person’s and partner’s SES in current year^a^	0.126 (0.115)	0.137 (0.103)	0.116 (0.106)	0.111 (0.109)	0.289* (0.112)	0.361** (0.113)	0.393*** (0.117)	0.279* (0.113)
(3) + Satisfaction with relationship	–0.054	–0.068	–0.074	–0.044	0.054	0.145	0.167	0.026
	(0.103)	(0.093)	(0.092)	(0.099)	(0.100)	(0.107)	(0.111)	(0.097)
Number of observations	924				1,127			

*Source:* Own calculations with UKHLS, HILDA, SOEP, and GGP.

^a^Childhood characteristics: region of origin, parent’s nativity, parental separation during childhood, mother’s and father’s education, mother’s and father’s employment status, and father’s occupational level. Partnership behavior: union duration, ever separated, and children within partnership. Respondent’s socioeconomic background in current year: educational level, employment status, household income, and self-rated health. Partner’s characteristics in current year: partner’s education and partner’s employment.

*p < .05; **p < .01; ***p < .001

Table 4 shows substantial differences by country, gender, and propensity to marry. In the United Kingdom, marriage is not associated with higher SWB for men relative to cohabitation. However, marriage seems to be, on average, more beneficial for women (ATE) (*p* < .05) and for partnered individuals with a low propensity to marry (ATC) (*p* < .01). These findings suggest that women with a high propensity to marry are just as happy regardless of whether they marry. Women with a low propensity to marry, however, seem to be happier if they marry. Contrary to expectations, including our large battery of controls increases the magnitude of the coefficients, thus implying that marriage becomes even more important, especially for those with a low propensity to marry. After relationship satisfaction is included, however, statistical differences between marriage and cohabitation are eliminated. This result may imply that the quality of the relationship matters more than whether it is legally recognized, or that only women with high-quality relationships and suitable marriage partners marry.

For Australian men, marriage is associated with higher SWB when only partnership status and age are included in the models. The level of significance is slightly higher for the unweighted and ATC (*p* < .01). Although all married people have higher levels of SWB than cohabitors, those with a lower propensity to marry would receive a slightly higher benefit if they married. However, when control variables are introduced, the coefficients in the weighted and unweighted models are no longer statistically significant, implying that selection processes matter in Australia. For Australian women, however, marriage provides benefits only in the unweighted and the ATC weighted regression models (*p* < .05); those with a low propensity to marry would be happier if they were to marry. However, significant differences between married and cohabiting women again disappear after controls are included.

For German women, marriage is not significantly different from cohabitation, suggesting that marriage does not provide additional SWB benefits over living with a partner. However, we find that before weighting, German married men have higher levels of SWB; after weighting, those living in a partnership with a low propensity to marry have higher levels of SWB (*p* < .01). After controls are introduced, though, the significant differences for men disappear, and the magnitude decreases. Even though the results show heterogeneity of treatment effects, they are surprising given our expectation of stronger differences between marriage and cohabitation in Germany.

Finally, for Norwegian men, as expected, marriage is not significantly different from cohabitation. For Norwegian women, however, all married women have higher levels of SWB compared with cohabiting women, regardless of the propensity to marry. Both those with a low propensity to marry and those with a high propensity to marry would experience benefits from marriage if they were to marry. Although control variables reduce the magnitude of the coefficients for those with a low propensity to marry (*p* < .05), they increase the magnitude and level of significance for those with a high propensity to marry (*p* < .001) and for the ATE (*p* < .001). This result is quite surprising because the Norwegian legal and social context suggests that marriage provides few advantages to SWB, and we would not expect that those with a high propensity to marry need to marry to be happy. However, we find that Norwegian women would have higher SWB if they married. Nonetheless, our analyses control for only socioeconomic characteristics and self-rated health; unobserved factors, such as personality or other psychological factors closely related to SWB, may be more important for decisions to cohabit or marry. Thus, although we can say that a large range of background characteristics do not eliminate differences by partnership type, we cannot truly ascertain a causal relationship for Norwegian women. In addition, an individual’s SWB may be dependent on the quality of the relationship with the partner. Including relationship satisfaction in the models eliminates differences between marriage and cohabitation, potentially implying that happy cohabiting relationships contribute just as much to SWB as high-quality marital relationships. On the other hand, Norwegian women may not be marrying because they have not found the right partner, which could be making them unhappy. Because relationship quality is measured only at the time of the survey, we cannot adjudicate between these explanations.

## Discussion

Prior studies examining the effects of SWB on partnership status have found that individuals receive a minor boost in happiness after moving in with a partner and a larger boost after marriage, although these effects generally wear off as married partners return to their set-point happiness (Kalmijn 2017; Musick and Bumpass 2012; Soons et al. 2009). What these studies cannot show is whether marriage is beneficial to those who are unlikely to marry, and the extent to which any marriage benefits are due to characteristics that select people into marriage. Prior studies have also not specifically focused on the effects of marriage relative to cohabitation in midlife, after most people have married, and the initial honeymoon period of marriage is over. Our study produced some surprising findings that indicate not only differences by country and gender but also differences by the propensity to marry.

First, contrary to prior studies (e.g., Ono and Lee 2012; Soons and Kalmijn 2009), our results indicate that relative to cohabitation, marriage does not automatically provide a boost to SWB in all countries. On average, cohabiting men in the United Kingdom and Norway and women in Germany have levels of SWB that are similar to those of married men in midlife, even without controls. These findings suggest that in some countries, cohabitation may provide benefits similar to those of marriage, such as shared intimacy, pooled resources, and emotional support (Musick and Bumpass 2012; Perelli-Harris and Styrc 2018).

Second, our results show that, on average, marriage does differ from cohabitation for Australian men and Norwegian women. Without any controls for selection, married individuals in these countries have higher levels of SWB than those in cohabiting partnerships. In Australia, these differences disappear after we include controls, indicating that cohabitation is selective of disadvantage, in accordance with prior studies (Evans 2015; Heard 2011). For Norwegian women, however, our entire battery of controls cannot eliminate average differences between cohabitation and marriage. This finding is quite surprising because some have argued that cohabitation and marriage in Norway, and Scandinavian countries in general, are indistinguishable (Heuveline and Timberlake 2004). Yet here, we see persistent differences between the two partnership types. Our models, however, take into account important sociodemographic status and childhood background variables but may be missing key psychological characteristics or other attributes that are associated with marriage. We cannot rule out the possibility that people with certain personality traits or preferences are more likely to marry. Thus, we are reluctant to interpret our results as having a causal effect.

Nonetheless, the findings suggest that marriage is more important in Norway than often assumed. Focus group research found that marriage is associated with romance and love, even if it occurs sometime after childbearing (Lappegård and Noack 2015), or as a capstone later in life (Holland 2017). Although cohabitation may seem to be identical to marriage superficially, marriage may be indicative of a closer partnership on a deeper level. Marriage for women may be symbolic of a more committed loving relationship, and if marriage does not happen by midlife, the lack of marriage might have detrimental effects on SWB. The elimination of marriage effects when we include relationship satisfaction suggests this may be the case. On the one hand, the results may indicate that relationship quality is more important than type of partnership, and cohabiting and married women have similar SWB. On the other hand, relationship satisfaction may instead be a proxy for marriage given that happier couples are generally more likely to marry (Wiik et al. 2009). Then again, the marital contract may in fact improve relationship quality for women. Thus, although we urge caution in interpreting this result one way or the other, it seems to be plausible that marriage, on average, has positive effects for women in Norway.

Third, our results demonstrate the heterogeneity of treatment effects for German men and British and Australian women. Partnered individuals who have a lower propensity to marry based on childhood selection mechanisms (ATC) have lower SWB if they cohabit rather than marry. Those who have a high propensity to marry (ATT), on the other hand, would not receive any benefits from marriage. For German men and Australian women, controlling for selection mechanisms and partnership experiences eliminates differences between cohabitation and marriage, again demonstrating that selection is more consequential for SWB than partnership status.

For British women, however, we find the persistent effect of marriage on SWB for those who are less likely to marry, despite the large number of controls. These results suggest that marriage may provide some benefits for disadvantaged women, as was also found in a study on mental well-being (Perelli-Harris and Styrc 2018). Our findings here, however, indicate that women from disadvantaged backgrounds would be better off if they did marry, but not because they or their partners have low education, poor employment conditions, or low income, which would make them unhappy. Instead, marriage seems to be associated with happiness for other reasons. The findings could be due to unobserved selection mechanisms related to personality, appearance, or other psychological factors that make them less-attractive marriage partners. On the other hand, they may be unhappy because they do not want to marry partners who do not live up to their expectations, or they may disagree about whether to get married, which could have a greater effect on women than men. The mediating effect of relationship quality suggests this may be the case; those who have higher-quality relationships marry and have higher levels of SWB. Qualitative research provides a deeper explanation for this finding: focus group participants from all educational levels in Britain generally agreed that marriage signaled a more committed relationship than cohabitation, but low-educated women stated that although they would like to marry, cohabitation was more common among their peers. For these women, marriage was a low priority compared with other responsibilities such as housing and children, but they nonetheless aspired to have a wedding (Berrington et al. 2015), and perhaps they were unhappier because they could not achieve their goals.

Our study is not without limitations. First, as mentioned, life satisfaction may be endogenous to partnership decisions: happier people may be more likely to marry than cohabit (Luhmann et al. 2013). Although we focus on controlling for selection mechanisms in childhood, before individuals enter into partnerships, our data do not include a direct measure of happiness in childhood or around the time of entrance into partnership. Propensity score–weighted regression is also unable to control for unobserved factors not available in our surveys. Marriage may be selective of other individual characteristics such as personality, emotional control, or attractiveness. This is particularly important for Norwegian and British women, where differences between cohabitation and marriage persist until relationship satisfaction is included in the models. Second, despite our concerted effort to harmonize the variables across our surveys, differences in survey design and variable construction may limit comparability across surveys. Finally, our definition of midlife (ages 38–50) is relatively narrow. But because cohabitation has increased only within the past few decades, the sample size for cohabiting individuals in the older cohorts is too small. Therefore, future research must continue to evaluate these relationships as cohabitation increases throughout midlife.

Despite these limitations, this study provides evidence that the relationship between partnership and SWB is not straightforward and is context-specific. The context, especially the policy context, does not always operate in predictable ways. For example, given that the German government privileges the marital breadwinner model, we would have predicted that married women in Germany would be happier than cohabiting women; however, we find no differences between marriage and cohabitation. This finding suggests that despite policies to encourage marriage, cohabitation in Germany may not be stigmatized; indeed, Treas et al. (2014) found that Germans have a more positive view of living in cohabitation without marriage intentions than those in Great Britain and Australia. Thus, the policy climate may not shape the association between partnership status and SWB as much as changing values and the specific meaning of cohabitation. This also holds true for Norwegian women, who (as discussed earlier) appear happier if they are married, despite a gender-friendly policy regime that stipulates few legal distinctions between cohabitation and marriage (Lappegård and Noack 2015). Regardless of an increasing number of children born in cohabitation and a lack of stigmatization toward cohabitors, Norwegian women still seem to value marriage and the symbolic implication of the wedding. Research on individual countries needs to recognize that context may be shaping the relationship between factors.

Finally, this study not only demonstrates the role of selection in accounting for the association between marriage and SWB but also illustrates how selection is heterogeneous and differs according to the propensity to marry. Such findings can have important policy implications given that those with a low propensity to marry—namely, the disadvantaged—are likely to be targeted by pro-marriage policymakers. At first glance, our findings seem to suggest that in some countries, those least likely to marry would benefit from marriage-promotion policies: if they were to marry, they would be happier. However, for German men and Australian women, the effect of marriage disappears after the inclusion of more controls. For disadvantaged women in the United Kingdom, marriage may matter, especially if it provides legal protection and a sense of security (Barlow 2014; Berrington et al. 2015). The effect in the United Kingdom disappears after a control for relationship satisfaction is included, implying that policymakers could focus on improving relationship quality, possibly through relationship support organizations that provide counseling, although it is also important to recognize that these women may be unhappy because they are unable to find a suitable partner. On the whole, however, our study indicates that especially after selection and relationship satisfaction are taken into account, differences between marriage and cohabitation disappear in all countries. Marriage does not cause higher SWB; instead, cohabitation is a symptom of economic and emotional strain. Thus, our findings imply that in order to increase SWB, policymakers should aim to reduce disadvantages—both in childhood and adulthood—instead of creating incentives to marry.

## Electronic supplementary material

ESM 1 (PDF 363 kb)

## Footnotes

1 “Happiness” and “life satisfaction” are often used interchangeably. *Life satisfaction* is typically defined as a process of assessing individuals’ perceived quality of life based on current circumstances (Diener et al. 2009). Here we use the term *subjective well-being* because it best reflects satisfaction with overall life.

2 We define *midlife* as aged 38–50. Older cohorts are less likely to be in a cohabiting partnership, and our sample becomes too small to analyze.

3 United Kingdom: “Please choose the number which you feel best describes how dissatisfied or satisfied you are with the following aspects of your current situation: Your overall life.” Australia and Germany: “All things considered, how satisfied are you with your life? Again, pick a number between 0 and 10 to indicate how satisfied you are.” Norway: “On a scale from 0 to 10, where 0 means ‘Not satisfied at all’ and 10 means ‘completely satisfied,’ how satisfied are you, on the whole, with your current life?”

4 A quadratic specification of union duration does not change the main results.

5 A respondent who has a child with a previous partner and the current partner are classified as having a child with the current partner.

6 We also ran models (available on request) that follow our theoretical framework and include each set of control variables separately: (1) childhood characteristics; (2) the respondent’s partnership and fertility behavior; and (3) socioeconomic background of the respondent and partner in the current year, which could be endogenous. These models did provide some nuances: for example, men’s own SES was particularly important for eliminating differences between cohabitation and marriage in Germany and Australia. Overall, however, the more detailed models do not change our main story. Therefore, we show tables with all control variables included at once.
