# Supplementary material for: Mind the “Happiness” Gap: The Relationship Between Cohabitation, Marriage, and Subjective Well-being in the United Kingdom, Australia, Germany, and Norway
Source: Demography. 2019 Jul 9;56(4):1219–46. doi: 10.1007/s13524-019-00792-4 (PMC6667403; doi:10.1007/s13524-019-00792-4)
Supplement: Supplementary file 1 — (PDF 363 kb) [file 13524_2019_792_MOESM1_ESM.pdf]

Online Appendix

Table A1: Logistic Regression Coefficients and Standard Errors for Prediction to be Married in Mid-life (38-50) for the UK, Australia, Germany, and Norway

	UK		AUS		GER		NOR	
	**Men**	**Women**	**Men**	**Women**	**Men**	**Women**	**Men**	**Women**
**Age**	0.044*	0.041*	0.932*	0.092*	0.053*	0.547	0.080*	0.127*
**Region[1]**								
- **1 (ref.)**								
- **2**	- 0.093	- 0.125	- 0.122	0.128	- 0.534*	- 0.463*	0.264	- 0.383
- **3**	- 0.281*	- 0.052	-	-	0.216	- 1.071*	0.688*	0.199
- **4**	- 0.206	- 0.031*	-	-	0.761*	1.057*	- 0.317	- 0.364
**Both parents are native**								
- **yes**	- 0.645*	- 0.457*	0.402*	0.334	- 0.051	- 0.034	0.653*	0.517
- **at least one foreign born (ref.)**								
**Parents lived together in childhood**								
- **yes**	0.651*	0.466*	0.585*	0.346	0.564*	0.446*	0.422	0.385
- **no (ref.)**								
**Mother's education**								
- **medium**	- 0.067	0.137	0.130	- 0.144	0.126	0.314*	- 0.029	0.273
- **high**	- 0.335	0.061	0.404	- 0.206	0.297	0.398*	0.047	0.137
- **low (ref.)**								
**Father's education**								
- **medium**	0.080	0.071	- 0.092	- 0.099	- 0.601*	- 0.723*	0.041	0.343
- **high**	0.184	0.125	0.036*	- 0.221	- 0.664*	- 0.745*	0.541	1.503*
- **low (ref.)**								
**Mother's employment**								
- **was employed**	- 0.191*	- 0.276*	- 0.088	- 0.025	- 0.435*	- 0.403*	- 0.049	0.142
- **was not employed (ref.)**								
**Father's employment**								
- **was employed**	- 0.095	0.209	- 0.052	0.190	- 0.054	0.264	0.181	0.061
- **was not employed (ref.)**								
**Father's occupation**								
- **medium**	0.239	0.232	0.247	0.130	- 0.073	0.020	0.238	- 0.465
- **high**	0.302*	0.266*	0.097	0.500*	- 0.257	0.263	0.172	- 0.258
- **low (ref.)**								
**Observations**	2744	3263	1262	1367	3341	3744	924	1127
**Model fit**								

Source: own calculations with UKHLS, HILDA, SOEP, GGP. * p<0.05

[1] Region: UK 1=South East, 2= Scotland + N. Ireland + North, 3= Midlands + Wales, 4 = South West, 4= ; GER 1= West, 2=born East + stayed East, 3= born East + moved West, 4= foreign born; AUS 1=Rural, 2= Urban; NOR

Table A2: Variable Balance between Treatment and Control Group Following Propensity-Score Weighting by Country

	UK		AUS		GER		NOR	
	**Men**	**Women**	**Men**	**Women**	**Men**	**Women**	**Men**	**Women**
**ATE Weight**								
Mean	0.02	0.02	0.02	0.03	0.04	0.03	0.03	0.03
SD	0.01	0.00	0.03	0.02	0.02	0.02	0.01	0.00
**ATT Weight**								
Mean	0.02	0.02	0.02	0.03	0.04	0.03	0.04	0.03
SD	0.01	0.00	0.03	0.02	0.02	0.02	0.02	0.01
**ATC Weight**								
Mean	0.01	0.01	0.01	0.01	0.02	0.02	0.02	0.02
SD	0.02	0.00	0.03	0.02	0.01	0.01	0.02	0.01

Source: **own calculations with UKHLS, HILDA, SOEP, GGS.**
Note: A value of 0 indicates perfectly balanced data.

Robustness Checks

Although we control for union duration, prior union separation, and joint children in our models, the cohabitors examined may not be exactly comparable to married people, who more often remain in first, long-lasting unions with children. Thus, we conducted sensitivity analyses restricted to: 1) those with children in relationships longer than three years, which is the typical period by which most cohabiting unions have transitioned to marriage or separated; and 2) those with children in first long-term relationships, i.e. those who never separated (models available on request). The results for those in long-term relationships with joint children were slightly different from the original models, but the story did not change by gender or country. For example, in the UK, marriage provided greater benefits to SWB on average for women, and the ATE and ATC coefficients were slightly higher after including controls. For German men, the differences between marriage and cohabitation before controls were greater, especially for ATE and ATT. For Australian men, differences for ATE and ATT were no longer significant, possibly due to reduced sample size. However, results were very similar for Norwegian women.

When examining the more restricted sample of those in first long-term relationships with children, the results only balanced for the UK and Australian samples. We found very similar results for British men and women compared to the full sample, except that women with a high propensity to marry (ATT) were happier if they married, when including all controls except relationship satisfaction. For Australian women, we found no differences between cohabiting and married women, unlike in the full sample. This may be because cohabitation is considered legally identical to marriage after six months or after having children, and the cohabiting couples in the restricted sample are more similar to their married counterparts, but it could also be due to reduced sample size. All in all, these robustness checks supported our main findings.

Supplemental Analyses

Analyses of partnered vs. unpartnered

An additional question emerging from our research is whether partnership in general, regardless of whether it is marital or cohabiting, is associated with higher levels of SWB. Table A3 shows that in all of our study countries, the magnitude of the coefficient for living with a partner is high and significant at the .001 level, when only age is included in the models. Nonetheless, controls do decrease the magnitude and reduce the level of significance considerably. In Australia, including controls completely eliminates differences between being partnered and unpartnered. For Norwegian men, the weighted regressions no longer show significant differences in SWB between those living with and without a partner; however the differences are maintained for Norwegian women. In the other countries, it does appear that those in a partnership do have higher levels of SWB, suggesting that partnership matters for well-being. However, we are reluctant to consider this a causal effect, considering our models are unable to account for many unobserved factors that could be important for SWB.

Table A3. OLS weighted regression of subjective well-being using propensity scores. Estimates show coefficients for the association between being in a partnership and subjective well-being relative to being single at mid-life (38-50 years old).

	**Un-weighted**	**ATE**	**ATT**	**ATC**	**Un-weighted**	**ATE**	**ATT**	**ATC**
	**Men**				**Women**			
**UK**								
(1) Partnered vs. unpartnered + age	0.993***	0.928***	0.915***	0.984***	0.864***	0.872***	0.820***	0.857***
(2) + Childhood characteristics + partnership behavior + person's & partner's SES in current year[1]	0.484***	0.434***	0.426***	0.465***	0.464***	0.496***	0.448***	0.503***
Observation numbers	4402				4539			
**Germany**								
(1) Partnered vs. unpartnered + age	0.865***	0.914***	0.924***	0.859***	0.829***	0.869***	0.874***	0.851***
(2) + Childhood characteristics + partnership behavior + person's & partner's SES in current year[1]	0.493***	0.460***	0.488***	0.328**	0.328***	0.350**	0.355**	0.332**
Observation numbers	3989				4841			
**Australia**								
(1) Partnered vs. unpartnered + age	0.458***	0.458***	0.462***	0.448***	0.578***	0.591***	0.600***	0.571***
(2) + Childhood characteristics + partnership behavior + person's & partner's SES in current year[1]	0.146	0.123	0.128	0.112	0.212	0.185	0.180	0.193
Observation numbers	1792				1995			
**Norway**								
(1) Partnered vs. unpartnered + age	0.381***	0.342***	0.330***	0.380***	0.608***	0.568***	0.555***	0.602***
(2) + Childhood characteristics + partnership behavior + person's & partner's SES in current year[1]	0.321*	0.258	0.254	0.270	0.507***	0.507***	0.496***	0.532***
Observation numbers	1218				1567			

Source: own calculations with UKHLS, HILDA, SOEP, GGP. Note: * p<0.05, **<0.01, *<0.001**

[1] Childhood characteristics: region of origin, parent's nativity, parental separation during childhood, mother's and father's education, mother's and father's employment status, father's occupational level; Partnership behavior: union duration, ever separated, children within partnership; Person's socio-economic background in current year: educational level, employment status, household income grouped in quintiles, self-rated health; Partner's characteristics in current year: partner's education, partner's employment.
